# Efficacy and safety of low‐dose sacubitril/valsartan in heart failure patients: A systematic review and meta‐analysis

**DOI:** 10.1002/clc.23971

**Published:** 2023-01-17

**Authors:** Wen‐Wen Chen, Juan Jiang, Jie Gao, Xiu‐Zhen Zhang, Yuan‐Min Li, Yan‐Lin Liu, He‐Qin Dang

**Affiliations:** ^1^ Department of Pharmacy The Second Affiliated Hospital of Shandong First Medical University Tai'an China; ^2^ Department of Stomatology The Second Affiliated Hospital of Shandong First Medical University Tai'an China; ^3^ Department of Cardiology The Second Affiliated Hospital of Shandong First Medical University Tai'an China

**Keywords:** dose, efficacy, heart failure, meta‐analysis, sacubitril/valsartan, safety

## Abstract

**Background:**

Controversy has persisted over the clinical benefits of low‐dose sacubitril/valsartan in patients with heart failure (HF).

**Hypothesis:**

Low‐dose sacubitril/valsartan might also be effective and safe in HF patients.

**Methods:**

Electronic databases including PubMed, Ovid, and Cochrane Library were systematically retrieved from inception to August 5, 2021. Review manager 5.4 and Stata 15.1 were employed in this systematic review and meta‐analysis. Key efficacy outcomes of interest included HF hospitalization, all‐cause mortality, left ventricular ejection fraction (LVEF), N‐terminal pro‐B‐type natriuretic peptide (NT‐proBNP), together with New York Heart Association (NYHA) functional class. The safety outcome was systolic blood pressure (SBP). The grading of recommendations assessment, development, and evaluation approach was conducted to evaluate the quality of evidence for each outcome.

**Results:**

A total of 1269 studies were screened and 9 real‐world studies met the inclusion criteria were included in the meta‐analysis, with 1697 participants. Compared with low‐dose sacubitril/valsartan, high‐dose sacubitril/valsartan significantly reduced the risk of HF hospitalization (odds ratio [OR]: 0.4, 95% confidence interval [CI]: 0.27–0.61, *p* < .0001) and the risk of all‐cause mortality (OR: 0.23, 95% CI: 0.11–0.47, *p* < .0001). However, there were no appreciable differences in improvements of NYHA (OR: 0.59, 95% CI: 0.15–2.35, *p* = .45), changes of LVEF (mean difference [MD]: 2.73%, 95% CI: −2.24% to 7.7%, *p* = .28), changes of NT‐proBNP (MD: 43.09, 95% CI: −28.41 to 114.59, *p* = .24) and changes of SBP (MD: 3.01, 95% CI: −4.62 to 10.64, *p* = .44) between groups with low‐dose and high‐dose sacubitril/valsartan.

**Conclusions:**

Compared with high‐dose sacubitril/valsartan, low‐dose sacubitril/valsartan was associated with increased risks of HF hospitalization and all‐cause mortality. However, no distinct between‐group differences in improvements of NYHA, changes of LVEF, changes of NT‐proBNP and changes of SBP were observed.

## 1 INTRODUCTION

Heart failure (HF), the most detrimental and costly disease, is a major public health problem affecting approximately 40 million adults globally.^1^, ^2^ In spite of guideline‐recommended optimal medical therapy, the prognosis of HF remains poor and the mortality and morbidity are still high in 5 years.^3^

As a novel treatment option for HF, sacubitril/valsartan showed a remarkable reduction in HF hospitalization (21%) and all‐cause mortality (16%) in the prospective comparison of angiotensin receptor neprilysin inhibitor with an angiotensin‐converting enzyme inhibitor to determine impact on global mortality and morbidity in HF (PARADIGM‐HF) trial.^4^ Similarly, real‐world beneficial effects of sacubitril/valsartan on an improvement in biomarkers, New York Heart Association (NYHA) functional class, and cardiac reverse remodeling, together with a reduction in hospitalization for HF were also described by several recent reports.^5^, ^6^, ^7^, ^8^

In particular, all selected patients in the PARADIGM‐HF trial were required to enter a single‐blind prerandomization run‐in phase to ensure they tolerated a prespecified maximum dose of sacubitril/valsartan. As a result, large proportion of patients (74.76%) achieved target dose of sacubitril/valsartan in the trial.^4^, ^9^ Indeed, it is well known from registry data, low dose of sacubitril/valsartan is very common in real‐world clinical setting due to several factors (symptomatic hypotension, hyperkalemia, renal dysfunction, and worsening HF), which was a clear difference from landmark trial.^6^, ^10^, ^11^, ^12^, ^13^ Two studies discovered that underdose of sacubitril/valsartan was associated with a higher risk of all‐cause death or HF hospitalization.^10^, ^14^ However, a retrospective cohort study found that reduced doses of sacubitril/valsartan did not increase risk of mortality or hospitalization compared with sacubitril/valsartan at the target dose.^12^ Meanwhile, another two real‐world studies confirmed that low‐dose sacubitril/valsartan significantly reduced N‐terminal pro‐B‐type natriuretic peptide (NT‐proBNP), induced beneficial cardiac reverse remodeling and improved clinical functional performance in HF patients without severe adverse effect.^6^, ^15^

Therefore, there are still controversies on the effect of low‐dose sacubitril/valsartan versus high‐dose sacubitril/valsartan for patients with HF, which might bring some difficulties in decision‐making regarding the choice of sacubitril/valsartan doses in the clinical management of HF patients. This systematic review and meta‐analysis were conducted to evaluate the efficacy and safety of low‐dose sacubitril/valsartan for HF patients based on real‐world data, by comparing with high‐dose sacubitril/valsartan in daily practice.

## 2 METHODS

### 2.1 Data source and quality assessment

This systematic review and meta‐analysis were presented according to the Preferred Reporting Items for Systematic Reviews and Meta‐Analyses (PRISMA).^16^ Electronic databases including PubMed, Ovid, and Cochrane Library were systematically searched with no linguistic restrictions from inception to August 5, 2021. The following index terms and their similar keywords were used in the electronic search: (1) “heart failure” AND (2) “sacubitril/valsartan” OR “entresto” AND (3) “dose” OR “dosing” OR “overdosing” OR “underdosing.” The inclusion criteria included: (a) studies that compared the low‐dose sacubitril/valsartan (mean daily dose < 200 mg) with high‐dose sacubitril/valsartan (mean daily dose ≥ 200 mg) in patients with HF; (b) follow‐up duration ≥ 3 months after the index day; and (c) available data on the key efficacy and/or safety outcomes that we want to explore. Studies that did not report the data were excluded. Two investigators (Juan Jiang and Jie Gao) independently reviewed the titles and abstracts to exclude those obviously irrelevant studies, and then the eligible studies were selected by full‐text evaluation following the inclusion and exclusion criteria. Additionally, the reference lists of included articles were also checked to identify other potentially eligible publications. Any disagreement was resolved by discussion. The Newcastle Ottawa‐Scale (NOS), designed for case–control study and cohort study, was used to assess the methodological quality of each selected study. The NOS is mainly comprised of three dimensions: selection of participants, comparability among groups, and outcome assessment, ranging from 0 to 9 points. Articles with 6 points and above were rated as high‐level quality.^17^ The grading of recommendations assessment, development, and evaluation (GRADE) approach was employed to evaluate the quality of each outcome.^18^ The clinical protocols of all included studies were approved by local ethics, and informed consent of patients was obtained.

### 2.2 Data acquisition and clinical outcomes

The baseline characteristics of studies and patients were extracted by two authors (Xiu‐Zhen Zhang and Yuan‐Min Li) independently, and the discrepancy was resolved through consensus. The following data were extracted from each included study: first author's name, year of publication, study design, location, number of participants, age, gender, ejection fraction, follow‐up duration, and mean daily dose in high‐dose group and low‐dose group. Additionally, we also tried to contact the authors if a study did not offer the necessary data. Key efficacy outcomes of interest included HF hospitalization, all‐cause mortality, left ventricular ejection fraction (LVEF), NT‐proBNP, together with NYHA functional class. The safety outcome was systolic blood pressure (SBP).

### 2.3 Statistical analysis

Review manager 5.4 and Stata 15.1 were adopted in this systematic review and meta‐analysis. The *χ* ^2^ test and the *I* ^2^ test were used to examine heterogeneity in the Review manager. Odds ratio (OR) and 95% confidence interval (CI) were calculated for the risk of HF hospitalization, the risk of all‐cause mortality, and the improvement of NYHA class. Weighted mean difference and 95% CI were calculated for the changes in LVEF, NT‐proBNP, and SBP. Sensitivity analysis was performed to seek the reason of heterogeneity by the “one‐study removed” method. In addition, the Egger's and Bgger's test, and also the visual inspection of funnel plots, were hired to assess publication bias. A two‐tailed *p* < .05 was considered statistically significant.

## 3 RESULTS

### 3.1 Search results and study characteristics

**Figure 1:**
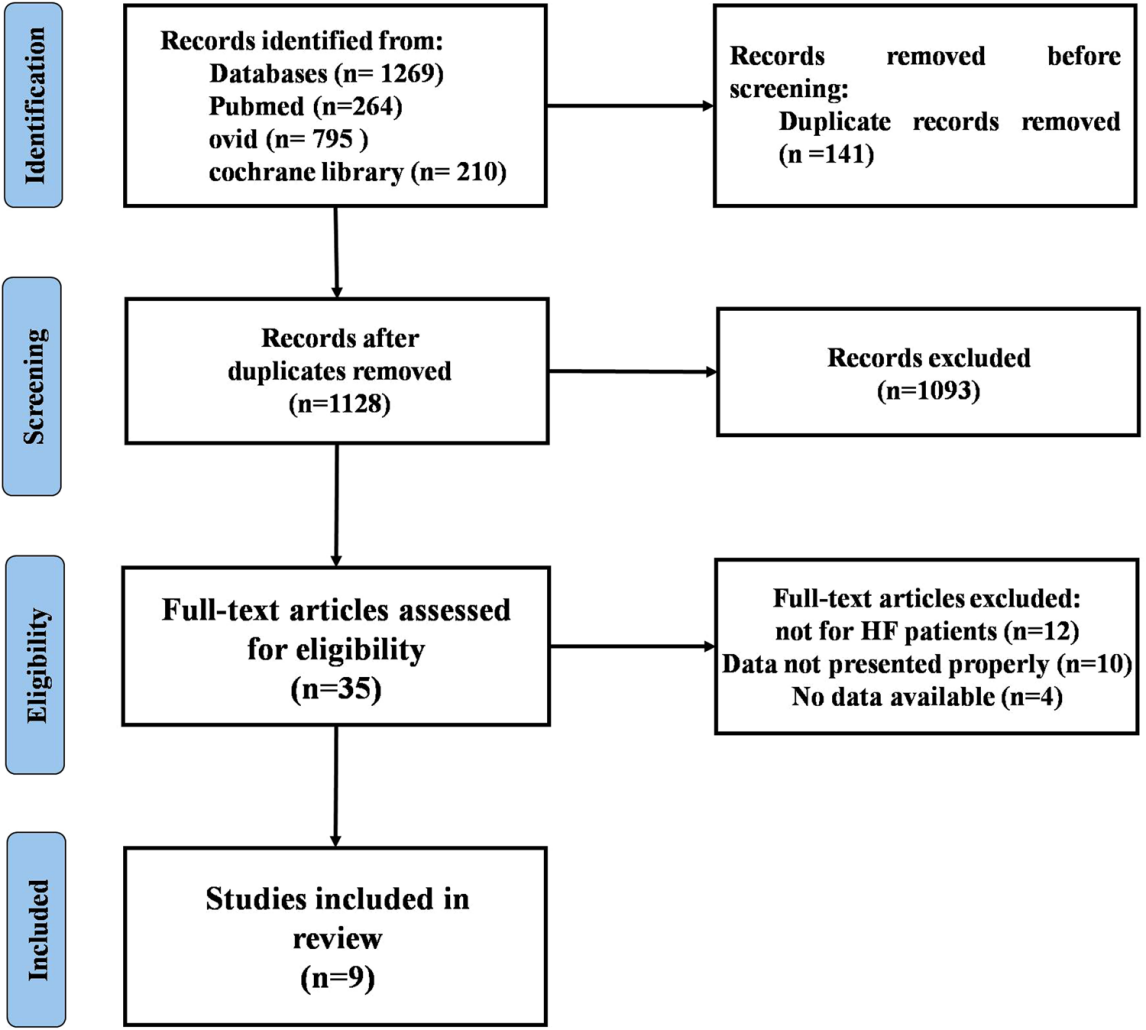
Flow diagram of literature search.

The process of literature screening and study selection is shown in Figure 1. Our search yielded a total of 1269 studies from PubMed, Ovid, and Cochrane library. After elimination of duplicate results, 1128 articles were reviewed. After exclusion of 1093 studies by title and/or abstract, 35 studies were reviewed in full‐text. Finally, nine real‐world studies met the inclusion criteria were included and analyzed in the meta‐analysis, with 1697 participants (Figure 1).

The characteristics of the included studies are summarized in Table 1. Three prospective and six retrospective studies were included in our meta‐analysis. Seven of nine studies were single‐center studies. As shown in Table 1, four studies were based in Europe, and the others were from China (2), America (1), Canada (1), and Australia (1). Mean age was from 59.2 ± 12.9 to 78.3 ± 6.9 years old, and 27.6% of participants were females. All patients of the studies that had been included in the meta‐analysis had LVEF of 40% or less, and mean LVEF ranged from 26.3 ± 8.6% to 35.4 ± 8.9%. The duration of follow‐up was 6.7 ± 3.2 months, ranging from 3 to 12 months. Mean high daily dose of sacubitril/valsartan ranged from 200 to 400 mg and the mean low daily dose of sacubitril/valsartan was from 87.7 to 175.3 mg. All the included studies had a high quality with a NOS score of ≥7 points (Supporting Information: Table 1).

**Table 1:** Characteristics of the included studies.

First author	Year	Type of study	Region	Participants	Age (years)	Female (%)	LVEF (%)	Follow‐up	High daily dose vs. low daily dose
Kido^10^	2021	Retrospective, multicenter study	America	721	65.2 ± 12.7	30.8	26.3 ± 8.6	12 months	400 vs. 167.8 mg
Almufleh^5^	2017	Retrospective, single center study	Canada	48	70 ± 11.1	20.8	26.4 ± 7.7	3 months	400 vs. 147.4 mg
Dashwood^19^	2020	Retrospective, single center study	Australia	209	59.2 ± 12.9	23.9	29.0 ± 9.5	6 months	400 vs. 175.3 mg
Vecchis^12^	2018	Retrospective, single center study	Italy	68	78.3 ± 6.9	51.5	37.1 ± 5.8	5 months	400 vs. 155 mg
Guerra^20^	2021	Prospective, multicenter study	Italy	226	64.3 ± 12.1	26.1	28.3 ± 5.6	6 months	400 vs. 100 mg
Hu^15^	2020	prospective, single center study	China	110	59.7 ± 13.3	22.7	35.4 ± 8.9	6 months	229.4 vs. 94.1 mg
Martens^21^	2018	prospective, single center study	Belgium	125	66 ± 10	19	29.6 ± 5.9	4 months	400 vs. 100 mg
Corrado^22^	2021	Retrospective, single center study	Italy	90	64.5 ± 10.9	17.8	30.0 ± 9.0	12 months	252.6 vs. 136.5 mg
Chen^8^	2021	Retrospective, single center study	China	100	62 ± 14	27	31.0 ± 6.0	6 months	200 vs. 87.7 mg

### 3.2 The efficacy outcomes

**Figure 2:**
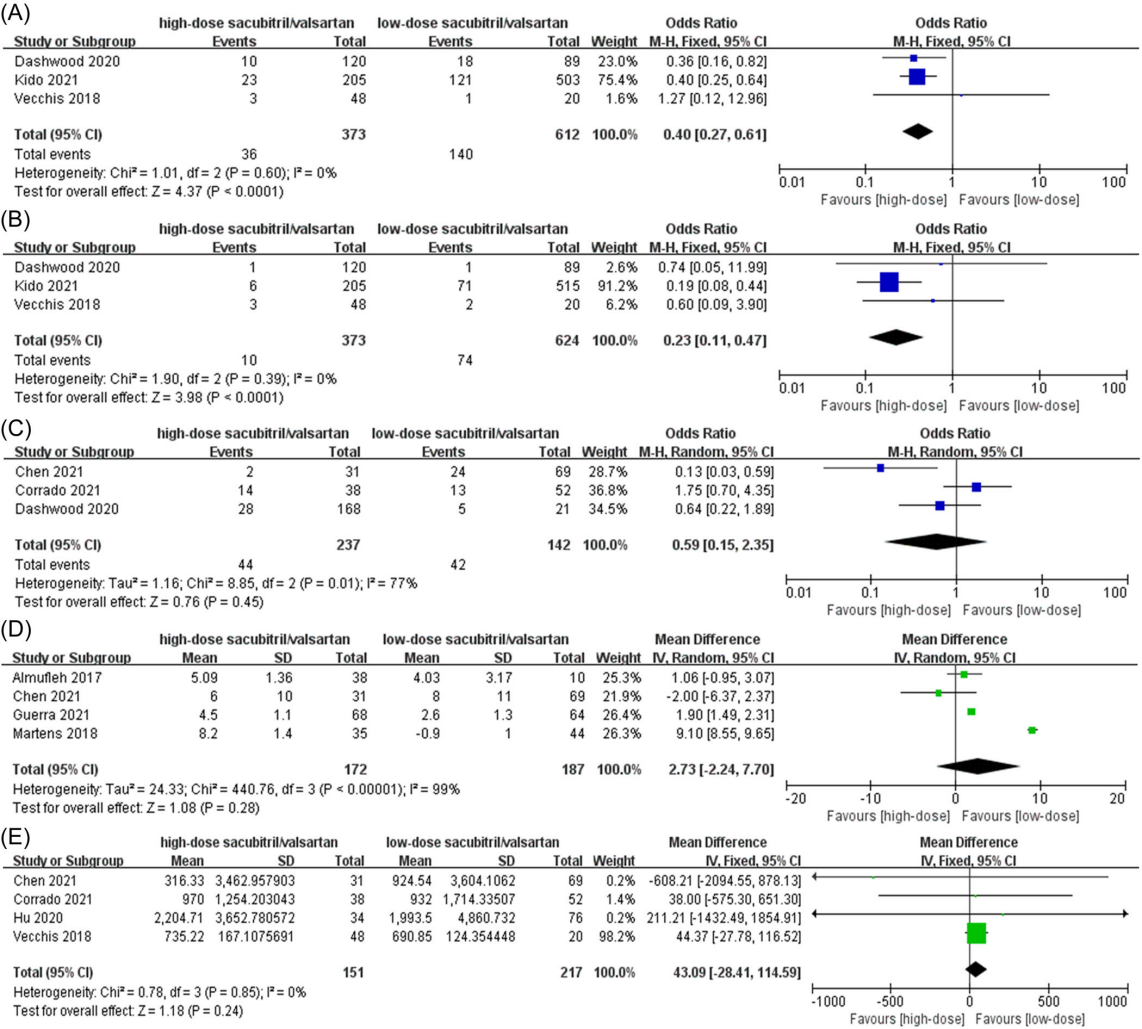
Comparison of efficacy endpoints between groups with low‐dose and high‐dose sacubitril/valsartan. (A) Heart failure hospitalization, (B) all‐cause mortality, (C) New York Heart Association, (D) left ventricular ejection fraction, (E) N‐terminal pro‐B‐type natriuretic peptide. CI, confidence interval; SD, standard deviation.

The HF hospitalization and all‐cause mortality were reported in three studies. Compared with low‐dose sacubitril/valsartan, high‐dose sacubitril/valsartan significantly reduced the risk of HF hospitalization (OR: 0.4, 95% CI: 0.27–0.61, *p* < .0001, Figure 2A) and the risk of all‐cause mortality (OR: 0.23, 95% CI: 0.11–0.47, *p* < .0001, Figure 2B) with no heterogeneity (*I* ^2^ = 0%). Improvements of NYHA were also presented in three trials. However, there were no obvious differences in the improvements of NYHA between groups with low‐dose and high‐dose sacubitril/valsartan (OR: 0.59, 95% CI: 0.15–2.35, *p* = .45, *I* ^2^ = 77%, Figure 2C). Changes of LVEF were shown in four research. The mean increase in LVEF was marginally greater in the high‐dose cohort as compared with the low‐dose cohort (mean difference [MD] 2.73%, 95% CI: −2.24% to 7.7%, *p* = .28, *I* ^2^ = 99%, Figure 2D). Of note, in a subset analysis of two studies that had largest contrast in daily dose (400 vs. 100 mg), there was also no significant difference in the mean increase in LVEF between patients with sacubitril/valsartan 400 mg daily and patients with sacubitril/valsartan 100 mg daily (MD: 5.5%, 95% CI: −1.56% to 12.55%, *p* = .13, *I* ^2^ = 100%, Supporting Information: Figure S1). Additionally, four trials mentioned the outcome of NT‐proBNP. No distinct differences in changes of NT‐proBNP were observed between patients with high‐dose sacubitril/valsartan and patients with low‐dose sacubitril/valsartan (MD: 43.09, 95% CI: −28.41 to 114.59, *p* = .24, *I* ^2^ = 0%, Figure 2E).

### 3.3 The safety outcomes

**Figure 3:**
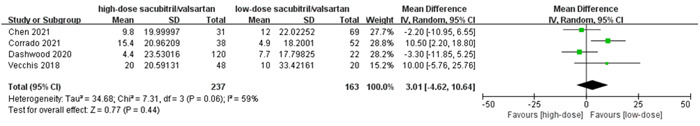
Comparison of changes of systolic blood pressure between low‐dose group and high‐dose group. CI, confidence interval; SD, standard deviation.

There were four studies evaluated the outcome of SBP. Changes of SBP in patients with high‐dose sacubitril/valsartan were similar to patients with low‐dose sacubitril/valsartan (MD: 3.01, 95% CI: −4.62 to 10.64, *p* = .44, *I* ^2^ = 59%, Figure 3).

### 3.4 Assessment of quality and publication bias

The quality was assessed. All the included studies had a high quality with an NOS score of ≥7 points (Supporting Information: Table 1). The quality assessments of GRADE evidence for each outcome were demonstrated in Supporting Information: Table 2. There was low evidence of HF hospitalization and all‐cause mortality, and very low evidence of LVEF, NT‐proBNP, NYHA, and SBP. No publication bias was detected in all outcomes. All studies included in the present meta‐analysis were symmetrically distributed in funnel plot (Supporting Information: Figure S2). The *p* value of the Begg's test and Egger's test were shown in Supporting Information: Table 3 (*p* > .05 for all).

## 4 DISCUSSION

This meta‐analysis demonstrated that high‐dose sacubitril/valsartan significantly reduced the risk of HF hospitalization and all‐cause mortality compared with low‐dose sacubitril/valsartan. However, there were no appreciable differences in improvements of NYHA, changes of LVEF, changes of NT‐proBNP, and changes of SBP between groups with low‐dose and high‐dose sacubitril/valsartan.

In the post hoc analysis of the PARADIGM trial,^23^ any dose reduction group receiving either enalapril or sacubitril/valsartan had a significantly higher event rate of all‐cause mortality or hospitalization for HF compared with the target‐dose group. However, due to enalapril mixed in the dose reduction group, the study could not provide a clear answer regarding whether there was an obvious difference in risk of HF hospitalization and risk of all‐cause mortality between the target‐dose sacubitril/valsartan group and the low‐dose sacubitril/valsartan group. Notably, our present study confirmed that the low‐dose sacubitril/valsartan was associated with a significantly higher event rate of all‐cause mortality or hospitalization for HF compared with the high‐dose sacubitril/valsartan. Unfortunately, evidence assessed by the GRADE method for those clinical outcomes (all‐cause mortality and HF hospitalization) was low. This was mainly because all studies reported all‐cause mortality and HF hospitalization included in the present meta‐analysis were observational studies. Nevertheless, the outcomes mentioned above were still acceptable. First, all the included studies had a high quality with a NOS score of ≥7 points. Secondly, no significant heterogeneity was found. Finally, no publication bias was detected by the funnel plot and the statistic test.

Apart from HF hospitalization and all‐cause mortality, changes in NYHA functional class, LVEF, and NT‐proBNP levels before and after initiation of sacubitril/valsartan were also assessed in many real‐world studies. Similar to other real‐world studies,^13^, ^24^, ^25^ the three studies included in the meta‐analysis also observed significant improvements in NYHA. Moreover, it was worthy of note that no obvious difference in NYHA improvement between the high‐dose group and the low‐dose group was found in the present meta‐analysis. The GRADE assessment found a very low quality of evidence and sensitivity analysis revealed that no significant between‐group difference in changes of the overall effect size (OR: 1.11, 95% CI: 0.41–2.96, *p* = .84) was observed with reduced heterogeneity (*I* ^2^ = 49%, *p*_heterogeneity_ = 0.16) after removing the study.

It is generally recognized that improvement of LVEF during treatment for HF is strongly associated with improved survival.^26^ Sacubitril/valsartan may favorably modify the trajectory of HF by improving LVEF.^27^ In the PROVE‐HF study, the 6‐ and 12‐month mean improvements in LVEF were 5.2% (95% CI: 4.8%–5.6%, *p* < .001) and 9.4% (95% CI: 8.8%–9.9%, *p* < .001) as compared with baseline, respectively. At 12 months, 75% of the study subjects had an LVEF increase of 4.9% or more and 25% experienced an LVEF increase of 13.4% or more.^28^ Unfortunately, the clinical trial did not investigate the effect of different doses of sacubitril/valsartan on the degree of improvement in LVEF. Based on real‐world data, our meta‐analysis observed no significant difference in changes of LVEF between high‐dose sacubitril/valsartan and low‐dose sacubitril/valsartan. Notably, the quality of evidence assessed by the GRADE method was very low due to significant heterogeneity, which may influence the applicability of the evidence. A sensitivity analysis was performed by the “one‐study removed” method, and one study that affected the overall effect size was identified. After excluding the results of this study, the heterogeneity was reduced (*I* ^2^ = 45%, *p*_heterogeneity_ = 0.16), but there was also no significant difference in changes of LVEF between the two groups (MD: 1.33%, 95% CI: −0.05% to 2.71%, *p* = .28).

Clinically, elevated levels of natriuretic peptides are useful for evaluating prognosis in HF, and hence a series of randomized controlled trials (RCTs)^29^, ^30^, ^31^ or real‐world studies^28^, ^32^ examined the effect of sacubitril/valsartan on NT‐proBNP. These research demonstrated similar results of reduction in NT‐proBNP. Notably, in an exploratory analysis of the efficacy and safety of sacubitril/valsartan according to dose level achieved in the PIONEER‐HF trial,^33^ the proportional reduction in NT‐proBNP concentration from baseline to Week 8 was consistent regardless of dose level (OR: 0.72, 95% CI: 0.58–0.88, *p* = .67). Similarly, our meta‐analysis observed that no significant differences in changes of NT‐proBNP between patients with high‐dose sacubitril/valsartan and patients with low‐dose sacubitril/valsartan. Although the quality of evidence for the outcome was very low, the conclusion was still acceptable. Because quality of all the included observational studies was high and no significant heterogeneity and publication bias were found.

With regard to the safety, real‐world studies demonstrated that hypotension occurred more frequently in patients receiving sacubitril/valsartan.^8^, ^34^, ^35^, ^36^ RCTs^29^, ^37^, ^38^, ^39^ and previous systematic reviews^40^, ^41^, ^42^ mainly investigated the effect of sacubitril/valsartan on blood pressure compared with standard therapy, but effect of different dose levels of sacubitril/valsartan on blood pressure reduction is unknown. Our meta‐analysis was the first to show that changes of SBP were consistent between high‐dose sacubitril/valsartan and low‐dose sacubitril/valsartan. Like the other outcome, the quality of evidence for changes of SBP was assessed as very low because of observational studies and heterogeneity. A sensitivity analysis was performed and one study that produced heterogeneity was identified. After excluding the study, the heterogeneity was reduced (*I* ^2^ = 10%, *P*_Heterogeneity_ = 0.33), but difference in changes of SBP between the two groups was still no significant (MD: −0.94, 95% CI: −7.03 to 5.15, *p* = .76).

### 4.1 Limitations

Some limitations should be acknowledged. First, real‐world observational studies with small sample sizes were employed in the present meta‐analysis to perform comparisons of efficacy and safety outcomes between high‐dose and low‐dose sacubitril/valsartan. This might contribute to the low quality of evidence assessed by GRADE approach for each outcome in this study, with implications for the reliability of the results of this meta‐analysis. In the future, the validity of this meta‐analysis needs to be confirmed by further RCTs with large sample sizes. Second, the high‐dose group and low‐dose group were classified according to the average daily dose of sacubitril/valsartan in the meta‐analysis. Therefore, the efficacy and safety of sacubitril/valsartan at the routine dosages (50 mg bid, 100 mg bid, and 200 mg bid) could not be assessed. Third, several efficacy (quality of life and exercise performance) and safety outcomes (renal function and hyperkalemia) could not be evaluated between high‐dose and low‐dose sacubitril/valsartan due to the small number of included studies and limited data, which needs to be further investigated. Fourth, due to patients with chronic kidney disease (CKD) mixed in all studies that had been included in the meta‐analysis, we could not achieve independent data to perform a subgroup analysis to explore if the effects of low‐dose sacubitril/valsartan were different in patients with CKD. Last, all patients of those studies that had been included in the meta‐analysis had LVEF of 40% or less. Therefore, we did not have sufficient data to allow for meaningful subgroup to explore if the efficacy and safety of low‐dose sacubitril/valsartan were different at different level of ejection fraction.

## 5 CONCLUSIONS

Compared with high‐dose sacubitril/valsartan, low‐dose sacubitril/valsartan was associated with increased risks of HF hospitalization and all‐cause mortality. However, no obvious differences in improvements of NYHA, changes of LVEF, changes of NT‐proBNP, and changes of SBP were observed in both study groups. These data showed that the clinical benefits of low dose of sacubitril/valsartan were not as good as those of high dose of sacubitril/valsartan. Additionally, since all the included studies were observational studies, level of evidence for every analysis assessed by GRADE approach was low and conclusions of the meta‐analysis should be applied with caution. Further RCTs with large sample sizes should be required to confirm the efficacy and safety of sacubitril/valsartan at different dose levels.

## AUTHOR CONTRIBUTIONS

**Wen‐Wen Chen**: Study design and manuscript. **Juan Jiang, Jie Gao, Xiu‐Zhen Zhang**, and **Yuan‐Min Li**: Data collection, data analysis, and validation. **Yan‐Lin Liu and He‐Qin Dang**: Scientific revision of the manuscript.

## CONFLICT OF INTEREST

The authors declare no conflict of interest.

## Supporting information

Supporting information.

Supporting information.

Supporting information.

Supporting information.

Supporting information.

## Data Availability

The data supporting this network meta‐analysis are from previously reported studies and datasets, which have been cited.
