# Supplementary material for: Efficacy and safety of low‐dose sacubitril/valsartan in heart failure patients: A systematic review and meta‐analysis
Source: Clin Cardiol. 2023 Jan 17;46(3):296–303. doi: 10.1002/clc.23971 (PMC10018087; doi:10.1002/clc.23971)
Supplement: Supplementary file 1 — Supporting information. [file CLC-46-296-s004.docx]

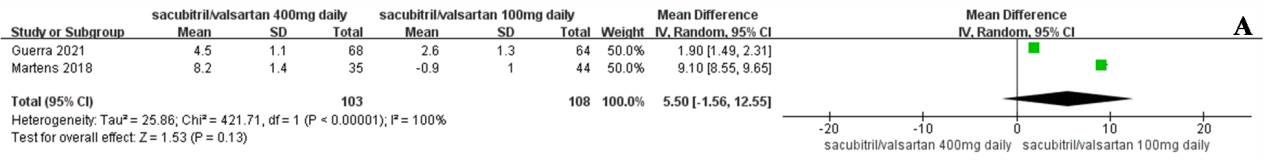


**
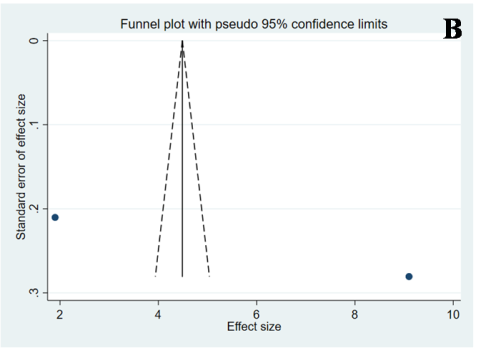
**

**Supplemental Figure S1.** Comparison of mean increase in LVEF between patients with sacubitril/valsartan 400mg daily and patients with sacubitril/valsartan 100mg daily. (A) Forest plot, (B) Funnel plot.
