# Supplementary material for: Efficacy and safety of low‐dose sacubitril/valsartan in heart failure patients: A systematic review and meta‐analysis
Source: Clin Cardiol. 2023 Jan 17;46(3):296–303. doi: 10.1002/clc.23971 (PMC10018087; doi:10.1002/clc.23971)
Supplement: Supplementary file 2 — Supporting information. [file CLC-46-296-s002.docx]

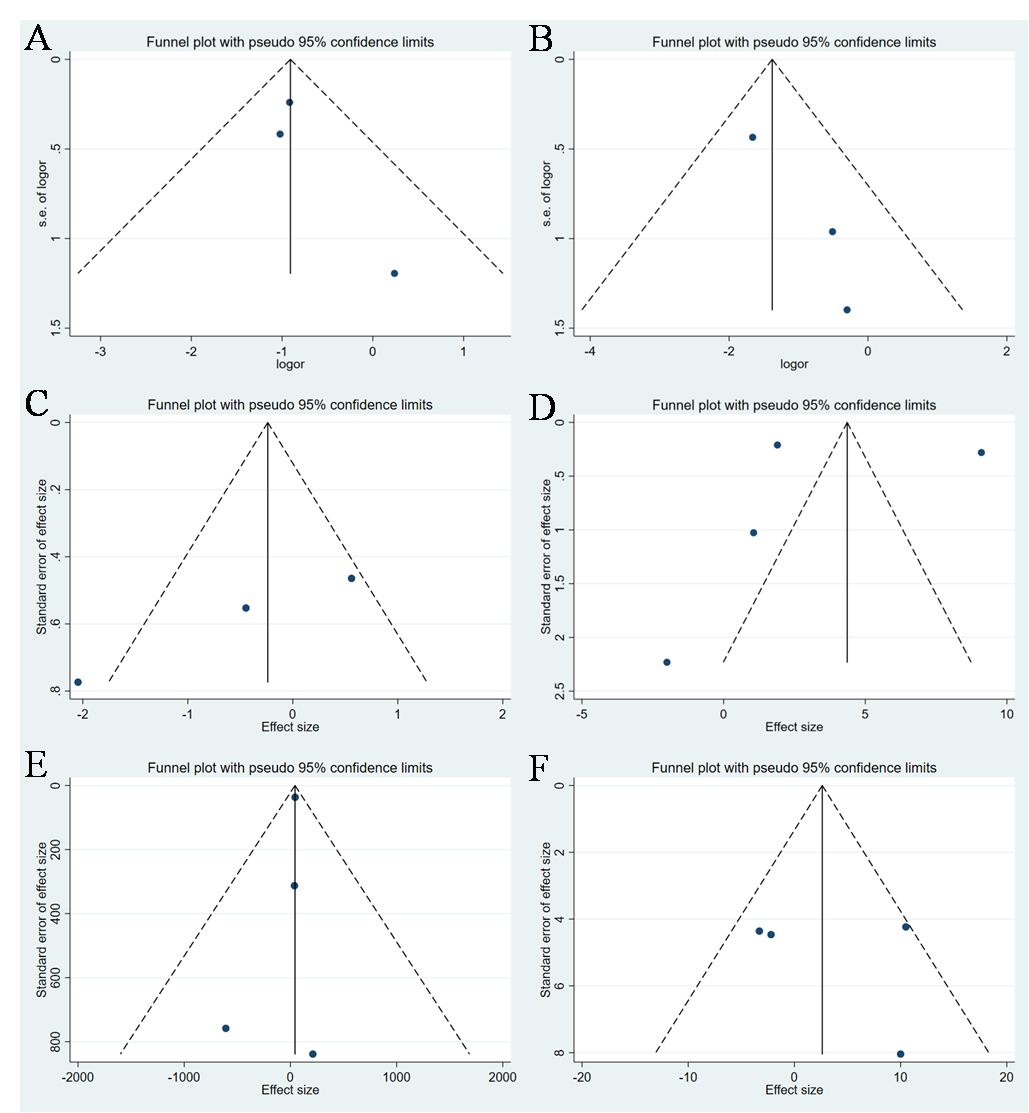


**Supplemental Figure S2.** The funnel plots of each outcome. (A) HF hospitalization, (B) all-cause mortality, (C) NYHA , (D) LVEF, (E) NT-proBNP, (F) SBP.
