# Supplementary material for: Efficacy and safety of low‐dose sacubitril/valsartan in heart failure patients: A systematic review and meta‐analysis
Source: Clin Cardiol. 2023 Jan 17;46(3):296–303. doi: 10.1002/clc.23971 (PMC10018087; doi:10.1002/clc.23971)
Supplement: Supplementary file 3 — Supporting information. [file CLC-46-296-s001.docx]

**Supplemental Table 1.** Quality assessment of the included studies based on the Newcastle-Ottawa Scale (NOS) items.

| Studies | Selection | | | | Comparability | Outcome | | | Total |
| --- | --- | --- | --- | --- | --- | --- | --- | --- | --- |
|  | Exposed  cohort | Non-exposed  cohort | Ascertainment  of exposure | Outcome  of interest |  | Assessment of  outcome | Length of  follow-up | Adequacy of  follow up |  |
| Kido-2021 | ***** | ***** | ***** | ***** | ***** | ***** | ***** | ***** | 8 |
| Almufleh  -2017 | ***** | ***** | ***** | ***** | ***** | ***** |  | ***** | 7 |
| Dashwood -2020 | ***** | ***** | ***** | ***** | ***** | ***** | ***** | ***** | 8 |
| Vecchis-2018 | ***** | ***** | ***** | ***** | ***** | ***** |  | ***** | 7 |
| Guerra-2021 | ***** | ***** | ***** | ***** | ***** | ***** | ***** | ***** | 8 |
| Hu-2020 | ***** | ***** | ***** | ***** | ***** | ***** | ***** | ***** | 8 |
| Martens -2018 | ***** | ***** | ***** | ***** | ***** | ***** |  | ***** | 7 |
| Corrado -2021 | ***** | ***** | ***** | ***** | ***** | ***** | ***** | ***** | 8 |
| Chen-2021 | ***** | ***** | ***** | ***** | ***** | ***** | ***** | ***** | 8 |
