# Supplementary material for: Efficacy and safety of low‐dose sacubitril/valsartan in heart failure patients: A systematic review and meta‐analysis
Source: Clin Cardiol. 2023 Jan 17;46(3):296–303. doi: 10.1002/clc.23971 (PMC10018087; doi:10.1002/clc.23971)
Supplement: Supplementary file 4 — Supporting information. [file CLC-46-296-s005.docx]

**Supplemental Table 2.** Quality assessments of GRADE evidence for each outcome.

| **Certainty assessment** | | | | | | | **№ of patients** | | **Effect** | | **Certainty** | **Importance** |
| --- | --- | --- | --- | --- | --- | --- | --- | --- | --- | --- | --- | --- |
| **№ of studies** | **Study design** | **Risk of bias** | **Inconsistency** | **Indirectness** | **Imprecision** | **Other considerations** | **Efficacy outcomes** | **placebo** | **Relative (95% CI)** | **Absolute (95% CI)** |  |  |
| **HF hospitalization** | | | | | | | | | | | | |
| 3 | observational studies | serious | not serious | not serious | not serious | dose response gradient | 36/373 (9.7%) | 140/612 (22.9%) | **OR 0.40**  (0.27 to 0.61) | - | ⨁⨁◯◯ Low | critical |
| **All-cause mortality** | | | | | | | | | | | | |
| 3 | observational studies | serious | not serious | not serious | not serious | dose response gradient | 10/373 (2.7%) | 74/624 (11.9%) | **OR 0.23**  (0.11 to 0.47) | - | ⨁⨁◯◯ Low | critical |
| **LVEF** | | | | | | | | | | | | |
| 4 | observational studies | serious | serious^a^ | not serious | not serious | strong association | 172 | 187 | - | MD **2.73% higher** (-2.24% lower to 7.7% higher) | ⨁◯◯◯ Very low | critical |
| **NT-proBNP** | | | | | | | | | | | | |
| 4 | observational studies | serious | not serious | not serious | not serious | none | 151 | 217 | - | MD **43.09 higher** (-28.41 higher to 114.59 higher) | ⨁◯◯◯ Very low | important |
| **NYHA** | | | | | | | | | | | | |
| 3 | observational studies | serious | serious^a^ | not serious | not serious | none | 62/237 (26.2%) | 42/142 (29.6%) | **OR 0.59**  (0.15 to 2.35) | - | ⨁◯◯◯ Very low | important |
| **SBP** | | | | | | | | | | | | |
| 4 | observational studies | serious | not serious | not serious | not serious | none | 237 | 163 | - | MD **3.01 higher** (-4.62 higher to 10.64 higher) | ⨁◯◯◯ Very low | critical |

CI: confidence interval; MD: mean difference; OR: odds ratio

a. There is significant heterogeneity (I2=99%).
