# Supplementary material for: Efficacy and safety of low‐dose sacubitril/valsartan in heart failure patients: A systematic review and meta‐analysis
Source: Clin Cardiol. 2023 Jan 17;46(3):296–303. doi: 10.1002/clc.23971 (PMC10018087; doi:10.1002/clc.23971)
Supplement: Supplementary file 5 — Supporting information. [file CLC-46-296-s003.docx]

**Supplemental Table 3.** The Begg’s and Egger’s Test in STATA of each outcome.

| Outcomes | P (Begg’s test) | P (Eegg’s test) |
| --- | --- | --- |
| HF hospitalization | 1.000 | 0.431 |
| all-cause mortality | 1.000 | 0.155 |
| LVEF | 0.734 | 0.963 |
| NT-proBNP | 0.734 | 0.547 |
| NYHA | 0.296 | 0.084 |
| SBP | 1 | 0.715 |
